# Application of an AI-enhanced interactive virtual clinical reasoning training system in orthodontic residency training

**DOI:** 10.3389/fmed.2026.1852507

**Published:** 2026-07-29

**Authors:** Qiannan Niu, Yi Wen, Qiuxiang Yuan, Yizhe Qi, Yuanqing Wu, Ying Yao, Jiaojiao He, Lei Wang, Fang Jin

**Affiliations:** 1 State Key Laboratory of Oral and Maxillofacial Reconstruction and Regeneration, National Clinical Research Center for Oral Diseases, Shaanxi Clinical Research Center for Oral Diseases, Department of Orthondontics, School of Stomatology, The Fourth Military Medical University, Xi'an, Shaanxi, China; 2 Key Laboratory of Hazard Assessment and Control in Special Operational Environment, Ministry of Education, Department of Military Health Statistics, Faculty of Military Preventive Medicine, The Fourth Military Medical University, Xi'an, Shannxi, China; 3 State Key Laboratory of Oral and Maxillofacial Reconstruction and Regeneration, National Clinical Research Center for Oral Diseases, Shaanxi Clinical Research Center for Oral Diseases, National Demonstration Center for Experimental Stomatology Education, School of Stomatology, The Fourth Military Medical University, Xi'an, Shaanxi, China

**Keywords:** artificial intelligence, case-based discussion, clinical reasoning, medical AI, medical student, orthodontics education

## Abstract

**Background:**

Case-based discussion is essential for developing clinical reasoning in orthodontics education, but its traditional format faces limitations in terms of case accessibility, teaching consistency, and scheduling flexibility. To address these challenges, we developed an AI-enhanced interactive virtual clinical reasoning training system and implemented it in our orthodontic curriculum. This study evaluates the educational effectiveness and practical utility of this innovative teaching system.

**Methods:**

A controlled trial was conducted with 74 residents who were randomly assigned to either an experimental group (*n* = 37) or a control group (*n* = 37). Both groups participated in case-based learning sessions on four common orthodontic cases. The experimental group used an AI-enhanced interactive virtual clinical reasoning training system, while the control group engaged in traditional small-group discussions. Teaching effectiveness was evaluated through objective examinations (theory test and case analysis) and an online questionnaire. System satisfaction was also assessed in the experimental group.

**Results:**

The experimental group achieved significantly higher case analysis scores and total scores than the control group (*p* < 0.001), although no significant difference was found in theory examination scores. In the teaching effectiveness survey, the experimental group rated all seven items significantly higher than the control group (*p* < 0.05). The system satisfaction survey indicated positive feedback across system functionality, skill improvement, and overall experience (mean scores: 4.35–4.57; Cronbach’s *α* = 0.919).

**Conclusion:**

The AI-enhanced interactive virtual clinical reasoning training system significantly improved residents’ case analysis performance and learning experience compared to traditional case-based learning. By simulating authentic clinical scenarios derived from real patient cases, the system bridges theoretical knowledge and clinical practice while fostering the development of humanistic competencies. This integrative approach offers a promising pathway for supporting medical students’ transition to clinical practice and enhancing case-based education in residency training.

## Introduction

Standardized residency training is a fundamental component of dental education in China and operates primarily within the “5 + 3” framework. This three-year programme is designed for five-year undergraduate graduates and professional degree postgraduate students. Through a structured and progressive training process, it facilitates the transition from a student to a practising dentist, aiming to comprehensively develop professional capabilities, specialized knowledge, patient care skills, communication and collaborative skills, the capacity for lifelong learning, and systematic clinical practice (1, 2, 3). At the heart of this professional development lies clinical reasoning. It serves as the foundational skill for diagnosing and managing oral health conditions, making it the essential conduit that transforms academic knowledge into effective patient care. Ultimately, cultivating this core competency in every dental trainee is a central mission of clinical teaching, as it not only defines expert practice but also drives progress in the field of dental science itself (4, 5).

In recent years, the implementation of standardized residency training has revealed several discipline-specific challenges in orthodontics (6). First, as undergraduate dental education seldom includes orthodontic internships, trainees typically encounter orthodontic patients for the first time during their residency. This delayed practical exposure often results in underdeveloped patient communication skills. Second, orthodontic cases are often clinically complex and highly variable and require adaptable treatment strategies and close interdisciplinary collaboration with fields such as general dentistry and clinical medicine. This level of complexity poses considerable difficulties for residents whose prior learning has been predominantly theoretical. Third, becoming a proficient orthodontist demands an extended training period supported by extensive clinical practice with a diverse range of patients. However, in many training institutions, the limited availability of patients restricts residents’ opportunities to translate theoretical knowledge into systematic clinical reasoning, hindering their progression from knowledge acquisition to qualitative professional development (7).

The rapid advancement of artificial intelligence (AI) has led to its increasing integration into clinical practice as a diagnostic support tool. Consequently, proficiency in utilizing AI has been proposed as a core competency for future medical graduates (8). Despite this recognized need, current medical education curricula offer limited instruction on how to critically integrate AI-generated diagnostic outputs into clinical reasoning and decision-making processes (9). Existing applications of AI in medical training, while diverse, remain fragmented. Examples include leveraging generative AI models such as ChatGPT for practicing patient-centered communication (10), applying AI-based diagnostic tools to enhance therapeutic decision-making in general practice clerkships (11), and employing AI within virtual reality environments for clinical and surgical skills training (12, 13). However, these initiatives largely consist of small-scale pilot studies or isolated module integrations (14). A cohesive, systematically designed educational framework that incorporates AI throughout the continuum of clinical training is still lacking. Therefore, this study developed an AI-enhanced interactive virtual clinical reasoning training system and applied it to case-based learning (CBL) in orthodontics.

The platform integrates a corpus of over 90 real-world clinical cases and constructs an immersive virtual diagnostic environment, enabling learners to engage in ubiquitous, self-directed training. The system features AI-assisted patient interviews, three-dimensional virtual physical examinations, and diagnostic and treatment planning modules, all designed to replicate the complexity of authentic clinical decision-making. Cases are stratified into three levels of difficulty based on clinical complexity and presentation, and the web-based interface supports both formative practice and summative assessment. By providing structured training on typical and complex cases, along with real-time feedback and process-oriented evaluation, the system aims to address the current deficiency in AI-integrated clinical education. Beyond the direct educational benefits, simulation-based training of this nature also serves a broader clinical purpose: it strengthens trainees’ clinical reasoning, team collaboration, and decision-making skills in a controlled, low-risk environment. Ultimately, this approach contributes to improved patient outcomes and safer, higher-quality care—a goal that lies at the heart of residency training (15). Furthermore, the system seeks to transition the role of AI from a passive instrument to an interactive collaborator in clinical reasoning, thereby laying the groundwork for a human-AI collaborative clinical reasoning framework in future medical practice.

## Platform functionality and educational applications

### Training approach during the residency programme

During our residency training programme, we implement a structured, phase-based educational approach that equally emphasizes theoretical learning and hands-on practice. The training process is organized as follows: Trainees first participate in small-group lectures that summarize key diagnosis and treatment principles for common malocclusions, helping them build a systematic theoretical foundation. They then engage in group discussions of actual cases, through which they gradually develop clinical reasoning skills specific to different types of malocclusions. Each resident subsequently completes training in the clinical environment. Finally, trainees undergo a comprehensive evaluation that includes both operational performance and results from a clinical reasoning assessment system. Those who meet the qualification standards are permitted to proceed to real clinical practice.

### AI-enhanced interactive virtual clinical reasoning training system in residency training

The interactive virtual clinical reasoning training system used in this study was powered by **DeepSeek-V4**, a large language model (LLM) developed by DeepSeek Inc. The model was integrated via a secure application programming interface (API) to enable three core functions: (1) natural language understanding for interpreting trainees’ free-text responses during simulated patient encounters; (2) dynamic clinical scenario generation, including patient history, physical examination findings, and diagnostic test results; (3) real-time formative feedback on clinical reasoning pathways. All data transmitted to and from the API were encrypted and de-identified in compliance with institutional data security policies.

To ensure the scientific rigor and reproducibility of the system, its development and validation process is described below. Ninety real clinical cases were sourced from the Department of Orthodontics at our institution, all de-identified and classified into beginner, intermediate, and advanced tiers. The teaching faculty collectively defined the selection criteria to ensure coverage across Angle Class I, II, and III malocclusions. A panel of three senior educators pre-defined and annotated the feedback logic, drawing on common reasoning errors observed during a pilot test with 15 residents. Each feedback entry specified the error type, offered a causal explanation, and provided actionable suggestions for improvement. The same pilot test served to validate and iteratively refine the feedback logic. To safeguard the accuracy of AI-generated feedback, the expert panel conducted sampling reviews, checking both clinical correctness and pedagogical soundness. The system’s knowledge base drew from standard orthodontic textbooks, domestic and international clinical guidelines, and the department’s own case library. Before full-scale educational deployment, the system underwent functional testing and process validation.

The system is built upon real clinical cases, which differ from simplified “standard cases” in their inherent complexity, frequent involvement of multidisciplinary considerations, and incorporation of critical decision-making variables such as patients’ subjective demands. The use of real clinical cases ensures that the training content maintains high fidelity to actual clinical scenarios that practitioners will encounter in the future (16, 17).

**Figure 1:**
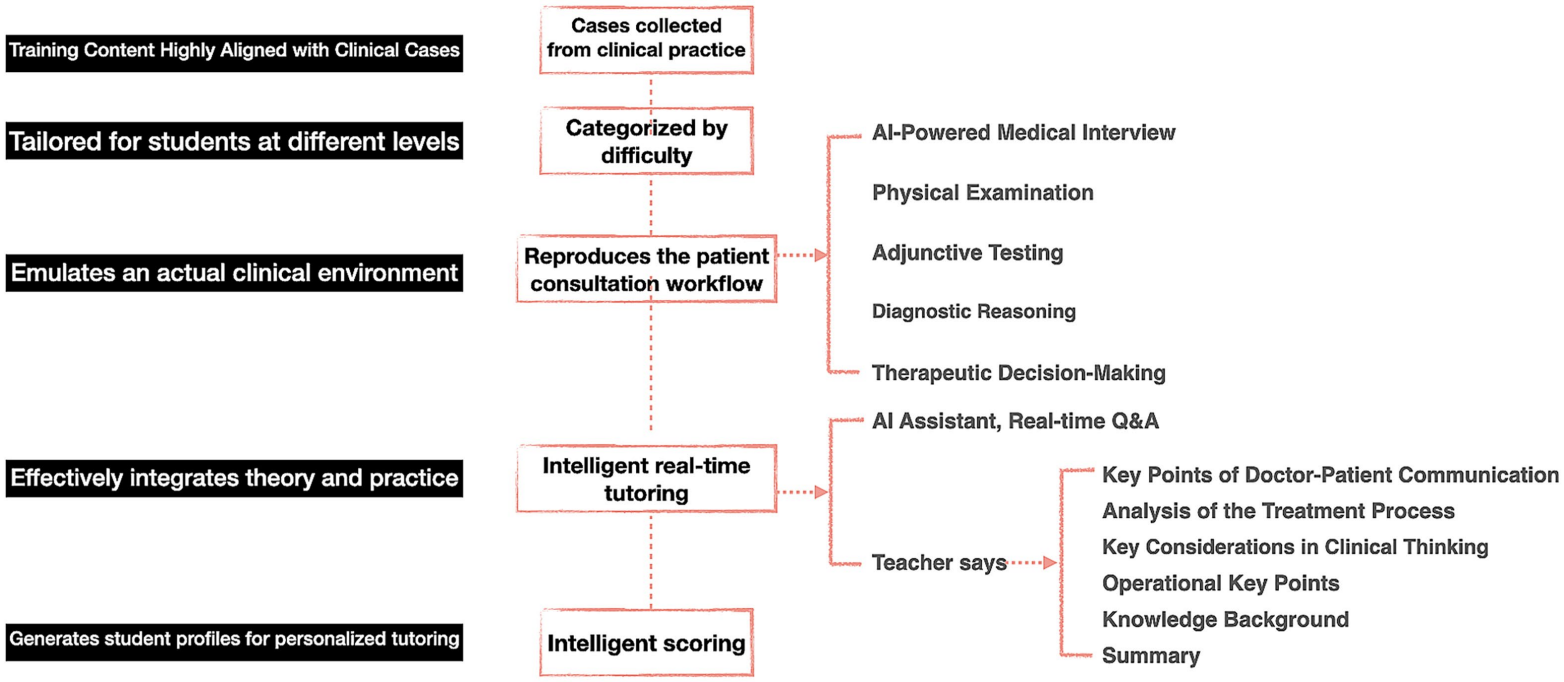
Design concept and rationale.

The design logic and workflow of the system are shown in Figure 1. This platform categorizes cases into three difficulty levels—corresponding to the first, second, and third years of residency—with each level containing 30 real clinical cases. Each level encompassing both common conditions and classic complex disorders. This structured design ensures that instruction strictly adheres to the medical education principle of “progressing from simple to complex, step by step,” providing trainees at different stages with clear, tailored and progressive learning paths.

The system simulates the entire clinical workflow, including AI-powered medical inquiry, oral examination, interpretation of auxiliary tests, and diagnosis and treatment planning, comprehensively covering key aspects of clinical practice. The AI medical inquiry module is specifically designed to address the challenge of cultivating and assessing competencies in doctor–patient communication and humanistic care—areas traditionally difficult to quantify in medical education (18). By simulating authentic clinical consultations, this component guides trainees to master essential orthodontic diagnostic skills through structured practice.

In terms of auxiliary examinations, the system supports cephalometric landmark analysis and the evaluation of panoramic radiographs and CBCT images and incorporates AI-based image analysis technology to provide intelligent recognition and diagnostic support. To further enhance the learning experience, the system features an integrated intelligence tutoring component. Its AI assistant, trained on the latest editions of authoritative foundational medical and dental textbooks used in China, offers users real-time, accurate theoretical knowledge support (19). The “Teacher says” section, curated by supervising instructors on the basis of actual patient consultations, includes key points for doctor–patient communication, critical thinking in clinical practice, operational key points, treatment process analysis, and case summaries, ensuring that students can thoroughly comprehend all aspects of each case (20).

The system is also built upon a scientifically grounded evaluation framework. Its most distinctive feature is the AI-powered consultation assessment, which transforms traditionally intangible “soft skills”—such as clinical inquiry, patient communication, and empathy—into structured, quantifiable metrics. For detailed scoring criteria and their respective weightings, please refer to Table 1. During the evaluation phase, the system also generates a personalized “student profile,” visualized through a radar chart, to display learning processes and outcomes in a scientifically intuitive manner. This visualization assists instructors in accurately discerning individual students’ competency characteristics, thereby providing both data-driven insights and practical means to move closer to the goal of truly personalized, adaptive teaching (21).

**Table 1:** AI consultation assessment criteria.

Scoring items	Sub-category	Item	Percentage
A. Information completeness	A1 Chief complaint and HPI	Chief complaint	10
		Onset and duration of illness	5
		Aetiology and predisposing factors	10
	A2 Past history	General health status	5
		Systemic diseases	5
		Other oral diseases	5
		Treatment history	5
		Allergy history	5
		Psychological status	5
	A3 Personal history	Occupation	5
		Lifestyle habits	5
		Adverse habits	10
	A4 Family history	Genetic oral diseases	5
B. Logic and efficiency of inquiry	B1 Consultation process	Questioning logic	5
	B2 Questioning efficiency	Validity and redundancy	5
C. Communication		Non-leading wording	5
		Absence of Professional Jargon	5

## Methods

The study was approved by the Institutional Review Board (IRB) at the Fourth Military Medical University ZL201425.

### Research object and methods

A total of 74 resident trainees who were enrolled in 2024 at the School of Stomatology, Fourth Military Medical University, participated in this study. A controlled trial design was employed. A total of 74 students were randomly divided into an experimental group (*n* = 37) and a control group (*n* = 37). Randomization was achieved by assigning each student a unique number from 1 to 74; those with numbers 1–37 were allocated to the experimental group, and those with numbers 38–74 to the control group. All participants provided informed consent before the study began.

### Teaching procedure

All students received identical instructional content and lecture hours, which were delivered by faculty members holding associate professor or higher academic titles from the same teaching department. Prior to instruction, all faculty participants engaged in collective lesson preparation to standardize teaching objectives. Each instructor involved in the study had successfully passed the National Resident Training Teaching Qualification Examination. The curriculum included 40 lectures and assigned readings from a major orthodontic textbook “Contemporary Orthodontics”, totaling 82 instructional hours.

For case-based discussion sessions, four common and representative orthodontic cases were selected following collective faculty preparation: early treatment of anterior crossbite, Class I malocclusion with crowding, Class II malocclusion, and Class III malocclusion. Two case discussion sessions were scheduled, each lasting 2 h, for a total of 4 h. The second session took place 1 week after the first. Both groups were taught by the same team of instructors during these case discussion sessions.

#### Teaching procedure of experimental group

Students in the experimental group were given access to the AI-enhanced interactive virtual clinical reasoning system 1 week before the class. During this preparation period, instructors also provided case summaries and key points for review. In class, students worked through cases independently using the system, then took part in group discussions. Each student completed the full clinical process within the system, from AI-assisted patient interviews and examinations to diagnosis and treatment planning. Throughout the session, instructors and teaching assistants tracked student progress using scores generated by the system, identified common difficulties, and offered real-time explanations and guidance as needed. After class, students could continue practicing on their own using the system, which included eight cases (two for each of the four case types) for additional practice.

#### Teaching procedure of control group

Students in the control group learned through a combination of traditional lectures and small-group discussions. One week before each session, they received case summaries, radiographic materials, and digital dental models, along with key learning points for preparation. In class, the sessions were mainly student-driven, with small-group discussions as the central activity. Instructors facilitated the process, helping students work through case examinations, auxiliary examinations, diagnoses, and treatment plans. Throughout the class, instructors observed group discussions and provided real-time guidance and clarification based on the difficulties students encountered. After class, eight additional cases (two for each of the four case types) were distributed to support continued self-directed learning.

### Evaluation of teaching effectiveness

Teaching effectiveness was evaluated from two perspectives: skills assessment and course satisfaction. Skills assessment included an objective structured examination and a self-administered online questionnaire. Course satisfaction was measured using a separate online questionnaire.

#### Skills assessment

The skills assessment consisted of an objective examination and an online questionnaire. The objective examination was administered 1 week after the case discussion sessions. Students in both groups took the same theory test and case analysis test designed by the instructors. The theory test had a maximum score of 70, while the case analysis test had a maximum score of 30. The case analysis section included three clinical scenarios. Based on the information provided, students were asked to propose appropriate examinations, diagnoses, and treatment plans, with particular emphasis on diagnosis and treatment planning. To ensure objectivity, each response was independently scored by three examiners, and the final score was calculated as the average of the three ratings.

The online questionnaire was completed anonymously at the end of the case discussions. It contained seven items, such as: “I remained highly focused and actively participated throughout the learning process,” “This teaching approach encouraged more independent thinking and clinical reasoning,” and “My ability to analyze and solve problems improved.” Responses were recorded on a 5-point Likert scale ranging from 1 (strongly disagree) to 5 (strongly agree). The questionnaire demonstrated good reliability, with a Cronbach’s *α* of 0.972 and split-half reliability coefficients of 0.964 and 0.965.

#### Satisfaction survey

A satisfaction survey was administered anonymously to students in the experimental group at the end of the case discussion sessions. The survey consisted of seven items addressing three areas: overall satisfaction, skill acquisition, and experience with system functions. Each item was rated on a 5-point Likert scale from 1 (strongly disagree) to 5 (strongly agree). Before the formal survey, a pilot version was tested with 20 randomly selected students. Items with factor loadings below the 0.7 threshold were revised based on pilot feedback and group discussion. The final version of the survey showed good internal consistency, with a Cronbach’s *α* of 0.919.

### Statistics

Statistical analysis was performed using SPSS for Windows, version 22.0. For continuous data, variables with a normal distribution were presented as mean ± standard deviation, while those with a skewed distribution results were expressed as median and interquartile range [M (IQR)]. Differences between groups were compared using the Mann–Whitney U test or the two-independent-sample t test, as appropriate. A *p* value of less than 0.05 was considered to indicate statistical significance.

The sample size was determined based on the expected effect size derived from prior educational intervention studies (Cohen‘s d = 0.7, considered a moderate-to-large effect). Assuming a two-sided significance level of 0.05, a statistical power of 0.80, and a 1:1 allocation ratio, G*Power software (version 3.1) indicated that a minimum of 34 participants per group would be required. To account for a possible 10% dropout rate, we set the target enrollment at 37 participants per group, for a total of 74 participants, which exceeded the estimated requirement.

## Results

### Comparison of baseline characteristics

Before implementing distinct training systems, statistical comparisons of participants’age, sex, and entrance examination score were conducted to confirm baseline characteristics between the two groups. Analysis revealed no statistically significant intergroup differences were observed for all baseline indicators (*p* > 0.05; Table 2).

**Table 2:** Baseline characteristics of study participants [*n*(%)/median (*P*_25_, *P*_75_) /x̄ ± s].

Variable	Experimental group	Control group	*χ*2/*U*/*t*	*P* value
Sex ^a^			0.237	0.626
Male	12 (32.4%)	14 (37.8%)		
Female	25 (67.6%)	23 (62.2%)		
Age ^b^	23.0 (23.0, 24.0)	24.0 (23.0, 24.0)	828.500	0.082
Entrance examination score ^c^	83.11 ± 1.807	83.49 ± 2.022	−0.849	0.399

a Chi-square test.

b Mann–Whitney U test.

c Independent-samples *t*-test.

### Comparison of theory examination scores

Following the different instructional interventions, students in both groups completed the theory examination. Statistical analysis showed that students in the experimental group achieved significantly higher case analysis scores and total scores compared to those in the control group (*p* < 0.001). Although the experimental group also scored slightly higher on the theory component, this difference did not reach statistical significance. Detailed results are presented in Table 3.

**Table 3:** Comparison of examination scores between the two groups.

Group	n	Theory examination score	Case analysis score	Total score
Experimental group	37	64.70 ± 1.431	25.16 ± 1.284	89.86 ± 2.069
Control group	37	64.27 ± 1.349	22.84 ± 1.027	87.11 ± 1.871
t value		1.756	7.728	5.744
*P* value		0.091	< 0.001	< 0.001

### Comparison of teaching effectiveness evaluations

A total of 74 questionnaires were distributed, and all were returned, yielding a response rate of 100%. All 74 questionnaires were valid and included in the analysis. Following the instructional intervention, students in the experimental group rated the teaching effectiveness significantly higher than those in the control group across all survey items. The differences between the two groups were statistically significant (all *p* < 0.05). Detailed results are presented in Table 4.

**Table 4:** Comparison of teaching effectiveness evaluations between the two groups M(IQR).

Group	n	Enhanced classroom engagement	Enhanced learning initiative	Enhanced knowledge application	Enhanced independent thinking	Enhanced problem-analysis and solving skills	Enhanced confidence
Experimental group	37	5(1)	5(1)	5(0)	5(1)	5(1)	5(1)
Control group	37	4(2)	4(2)	4(1)	4(2)	4(2)	4(2)
U value		986.50	942.50	1001.50	1002.50	1024.0	973.50
*P* value		< 0.001	0.002	< 0.001	< 0.001	< 0.001	< 0.001

### Experimental group’s evaluation of the new teaching method

A total of 37 questionnaires were distributed to students in the experimental group, and all were returned, yielding a response rate of 100%. The evaluation results regarding the teaching process are as follows. Students rated the following items with a median score of 5 (interquartile range, 1): the novelty of the teaching method attracted their attention; the AI-assisted inquiry module helped them connect previously learned knowledge; it improved their clinical communication skills; this teaching method helped me improve my case analysis and diagnostic skills; they expressed willingness to continue using this teaching method in subsequent learning; the interaction during the AI-assisted inquiry was smooth; and the AI assistant effectively supported the learning process. Overall, students reported high levels of satisfaction across all dimensions, including overall satisfaction, skill acquisition, and system functionality.

## Discussion

In this study, students in the experimental group achieved significantly higher scores in case analysis and overall academic performance compared to those in the control group. Within the context of this short-term educational intervention, this result suggests that the AI-enhanced interactive virtual clinical reasoning training system may help address several longstanding limitations associated with conventional case-based learning. Although traditional CBL is widely recognized for its role in developing clinical reasoning and collaborative skills (16–19), its effectiveness is often limited by factors such as restricted case variety, inconsistent teaching quality, and inflexible scheduling—all of which can hinder students from reaching their full clinical potential.

While artificial intelligence has received growing attention in medical education, most current applications remain focused on isolated skill areas. These include the use of generative AI for communication training (20–22), AI-assisted tools for image interpretation (23, 24), and virtual reality platforms for technical skill development (25–27). What has been lacking is a cohesive educational model that integrates AI across the entire clinical reasoning process—from patient interview to diagnosis and treatment planning. The system presented in this study fills this gap by embedding AI support throughout the case-based learning workflow. Through dynamic case generation, real-time feedback, and structured assessment of complex competencies such as empathy and doctor-patient communication, the system shifts AI’s role from a passive tool to an active contributor in the learning process. Within the context of this short-term educational intervention, this integrated approach may explain the improved clinical reasoning performance observed in the experimental group. However, given that this study measured immediate post-intervention outcomes rather than long-term clinical competence or real-patient performance, these findings should be interpreted as preliminary evidence of the system’s short-term educational effectiveness in case analysis and student learning experience.

It is worth noting that while the experimental group showed significant improvement in case analysis—a task requiring knowledge integration and application—their theoretical examination scores were not significantly different from those of the control group. This finding is consistent with the design purpose of the system, which emphasizes the application of knowledge in simulated clinical contexts rather than the memorization of factual content. Theoretical examinations, by contrast, primarily assess foundational knowledge and recall. This pattern reflects the system’s specific educational value: it appears to enhance higher-order cognitive skills without directly affecting rote memorization, positioning it as a complement to—not a substitute for—traditional didactic teaching.

In addition to the improvement in objective test scores, this study also found positive results in how students viewed their learning experience. Students in the experimental group gave higher ratings than those in the control group across all survey items related to teaching effectiveness. These differences were statistically significant (*p* < 0.05). The items covered classroom engagement, learning initiative, ability to apply knowledge, independent thinking, problem-solving skills, and confidence in learning. These findings suggest that the AI-assisted clinical reasoning system did more than just improve test performance. It also was associated with enhanced student-reported learning experience.

Compared with traditional teaching methods, the system may better engage students by simulating real clinical situations, offering immediate feedback, and allowing learners to progress at their own pace (28, 29). Two features appeared to be particularly helpful. The first was the interactive design of the AI-assisted inquiry module. The second was the “Master’s Forum,” where clinical experience was presented in a structured way. Together, these features helped students better understand how clinical decisions are made and gave them more confidence in their own skills.

The satisfaction survey completed by students in the experimental group looked at three areas with seven items in total. The skill acquisition area had three items, the system functionality experience area had two items, and the overall satisfaction area had two items. Mean scores for these three areas fell between 4.35 and 4.57, showing that students generally viewed the new teaching method in a positive light. One item in particular, “enhanced problem-solving skills,” received high ratings. This was consistent with the improvement seen in case analysis scores from the objective examination, suggesting that students’ self-reported gains matched their actual performance.

Student feedback also pointed to the value of the AI inquiry and three-dimensional examination tools. These features helped create realistic clinical situations and offered a way to practice doctor-patient communication—an area often difficult to teach and assess through traditional methods (30). Overall, the subjective evaluations and objective results were consistent with each other. Both point to the system’s potential utility in helping students develop aspects of clinical reasoning within the educational setting. The positive learning experience and increased confidence observed in this study are especially important for residents, as these factors can support continued motivation and professional growth during training.

The main contribution of this study is the development of an educational model that integrates AI into the full process of clinical reasoning within a case-based learning framework. Unlike most current uses of AI in medical education, which tend to focus on isolated skills, this system covers the entire case discussion process—from patient interview to diagnosis and treatment planning. In doing so, it offers a proof-of-concept for addressing a gap in current teaching practice related to AI-assisted clinical decision-making (22–27). The system generates dynamic cases, provides real-time feedback, and assesses complex skills such as empathy and communication in a structured way. By doing so, it shifts the role of AI from a passive tool to an active participant in learning. This represents a novel instructional approach for helping residents practice clinical reasoning skills in a simulated environment.

From a theoretical standpoint, this study offers early evidence that may support a “human-AI collaborative” approach to clinical reasoning as an educational strategy. In traditional settings, clinical reasoning is taught mainly through instructor guidance and the sharing of experience-based knowledge (31). The results of this study suggest that AI systems can, to some extent, take on a role similar to that of a clinical instructor in guiding students through the reasoning process. They can help students see how information is gathered and how decisions are made. In addition, the system translates abstract concepts like humanistic literacy and communication into measurable indicators. This provides a new way to cultivate and assess these important but often hard-to-measure skills (32). From a practical perspective, the system’s case library and structured teaching workflow are easy to adapt. Other institutions and disciplines may find it a useful reference for AI-assisted teaching. It also offers a practical solution for integrating digital tools into clinical reasoning training within residency programs and warrants further investigation in broader educational contexts.

This study has several limitations. First, the sample came from a single institution and was relatively small. This may limit how far the findings can be generalized. Second, the questionnaires used were developed by the research team based on the specific context of the study. Although they showed good internal consistency, their validity needs further testing. Third, the intervention was short. Only immediate outcomes were measured. It remains unclear whether the learning gains persist over time or whether they transfer to real clinical settings. Fourth, we did not record the specific preparation time for either group. However, given that both groups had parallel preparation periods and the difference in learning modality reflects the instructional approach being tested, this limitation does not affect the core conclusions. Fifth, the system itself can be improved. Possible enhancements include adding more cases, offering richer feedback in the AI inquiry module, and including tasks that require students to explain diagnoses and treatment plans to patients. Such additions could help develop empathy more fully. The user experience could also be improved. For example, adding AI-guided teaching features or enabling online group discussions could make the system more flexible and engaging.

Future studies should address these limitations. Multi-center trials with larger and more diverse samples are needed to test the system in different educational settings. Standardized assessment tools with proven reliability and validity should be used. Longitudinal designs would help determine whether the system has lasting effects on clinical competence. The system itself should continue to evolve. Integrating new technologies such as augmented reality or advanced natural language processing could make it more immersive and intelligent. With these efforts, the value of this AI-assisted clinical reasoning system can be further tested and extended—both in orthodontic education and in other areas of medical training.

## Conclusion

We developed an AI-enhanced interactive virtual clinical reasoning training system and tested it in case-based learning sessions with orthodontic residents. Students who trained with the system performed better on case analysis and reported more favorable learning experiences than those who learned through traditional small-group discussions. These results suggest that embedding AI support across the clinical reasoning workflow—from patient interview to treatment planning—can improve short-term case analysis skills and learner engagement in residency education. However, the study measured immediate outcomes only; we did not assess long-term retention or performance with real patients. Further work is needed to determine whether these short-term gains translate into sustained clinical competence.

## Data Availability

The original contributions presented in the study are included in the article/Supplementary material, further inquiries can be directed to the corresponding authors.
