# Supplementary material for: Application of an AI-enhanced interactive virtual clinical reasoning training system in orthodontic residency training
Source: Front Med (Lausanne). 2026 Jul 29;13:1852507. doi: 10.3389/fmed.2026.1852507 (PMC13463153; doi:10.3389/fmed.2026.1852507)
Supplement: Supplementary file 1 [file Table_2.DOCX]

1、I am able to maintain a high level of concentration and active participation during the learning process

□1 point（Strongly Disagree） □2 point（Disagree） □3 point（Neutral） □4 point（Agree） □5 point（Strongly agree）

2、I am willing to spend more time learning and exploring related content.

□1 point（Strongly Disagree） □2 point（Disagree） □3 point（Neutral） □4 point（Agree） □5 point（Strongly agree）

3、This teaching method has strengthened my ability to apply orthodontics.

□1 point（Strongly Disagree） □2 point（Disagree） □3 point（Neutral） □4 point（Agree） □5 point（Strongly agree）

4、This system has stimulated more independent thinking and clinical reasoning in me.

□1 point（Strongly Disagree） □2 point（Disagree） □3 point（Neutral） □4 point（Agree） □5 point（Strongly agree）

5、My ability to analyze and solve problems has improved.

□1 point（Strongly Disagree） □2 point（Disagree） □3 point（Neutral） □4 point（Agree） □5 point（Strongly agree）

6、I am confident in my ability to transition smoothly into clinical work.

□1 point（Strongly Disagree） □2 point（Disagree） □3 point（Neutral） □4 point（Agree） □5 point（Strongly agree）

、
