# Supplementary material for: Application of an AI-enhanced interactive virtual clinical reasoning training system in orthodontic residency training
Source: Front Med (Lausanne). 2026 Jul 29;13:1852507. doi: 10.3389/fmed.2026.1852507 (PMC13463153; doi:10.3389/fmed.2026.1852507)
Supplement: Supplementary file 2 [file Table_3.DOCX]

1、The novelty of the teaching method attracted their attention

□1 point（Strongly Disagree） □2 point（Disagree） □3 point（Neutral） □4 point（Agree） □5 point（Strongly agree）

2、the AI-assisted inquiry module helped them connect previously learned knowledge

□1 point（Strongly Disagree） □2 point（Disagree） □3 point（Neutral） □4 point（Agree） □5 point（Strongly agree）

3、it improved their clinical communication skills

□1 point（Strongly Disagree） □2 point（Disagree） □3 point（Neutral） □4 point（Agree） □5 point（Strongly agree）

4、this teaching method helped me improve my case analysis and diagnostic skills

□1 point（Strongly Disagree） □2 point（Disagree） □3 point（Neutral） □4 point（Agree） □5 point（Strongly agree）

5、they expressed willingness to continue using this teaching method in subsequent learning

□1 point（Strongly Disagree） □2 point（Disagree） □3 point（Neutral） □4 point（Agree） □5 point（Strongly agree）

6. the interaction during the AI-assisted inquiry was smooth

□1 point（Strongly Disagree） □2 point（Disagree） □3 point（Neutral） □4 point（Agree） □5 point（Strongly agree）

7. the AI assistant effectively supported the learning process

□1 point（Strongly Disagree） □2 point（Disagree） □3 point（Neutral） □4 point（Agree） □5 point（Strongly agree）
